# Shotgun metagenomic data of microbiomes on plastic fabrics exposed to harsh tropical environments

**DOI:** 10.1016/j.dib.2020.106226

**Published:** 2020-08-24

**Authors:** Osman Radwan, Oscar N. Ruiz

**Affiliations:** a Environmental Microbiology Group, University of Dayton Research Institute, Dayton, OH, USA; b Fuels and Energy Branch, Aerospace Systems Directorate, Air Force Research Laboratory, Wright-Patterson AFB, OH**,** USA

**Keywords:** Metagenomics, Microbiome, Bioinformatics, High-throughput sequencing, Biodegradation, Plastics

## Abstract

The development of more affordable high-throughput DNA sequencing technologies and powerful bioinformatics is making of shotgun metagenomics a common tool for effective characterization of microbiomes and robust functional genomics. A shotgun metagenomic approach was applied in the characterization of microbial communities associated with plasticized fabric materials exposed to a harsh tropical environment for 14 months. High-throughput sequencing of TruSeq paired-end libraries was conducted using a whole-genome shotgun (WGS) approach on an Illumina HiSeq2000 platform generating 100 bp reads. A multifaceted bioinformatics pipeline was developed and applied to conduct quality control and trimming of raw reads, microbial classification, assembly of multi-microbial genomes, binning of assembled contigs to individual genomes, and prediction of microbial genes and proteins. The bioinformatic analysis of the large 161 Gb sequence dataset generated 3,314,688 contigs and 120 microbial genomes. The raw metagenomic data and the detailed description of the bioinformatics pipeline applied in data analysis provide an important resource for the genomic characterization of microbial communities associated with biodegraded plastic fabric materials. The raw shotgun metagenomics sequence data of microbial communities on plastic fabric materials have been deposited in MG-RAST (https://www.mg-rast.org/) under accession numbers: mgm4794685.3–mgm4794690.3. The datasets and raw data presented here were associated with the main research work “Metagenomic characterization of microbial communities on plasticized fabric materials exposed to harsh tropical environments” (Radwan et al., 2020).

## Specifications Table

**Table:** 

Subject	Environmental Science
Specific subject area	Environmental Microbiology and Metagenomics
Type of data	Tables, Figures
How data were acquired	Illumina HiSeq2000 instrument was used for high-throughput sequencing of six genomic DNA libaries.
Data format	Raw and Analyzed
Parameters for data collection	Qiagen DNeasy UltraClean Microbial extraction kit (Cat# 12224-250) was used for DNA extraction from the six fabric materials for library preparation and DNA sequencing.
Description of data collection	A high-throughput sequencing of TruSeq paired-end libraries was conducted using whole-genome shotgun (WGS) approach on an Illumina HiSeq2000 platform generating 100 bp reads.
Data source location	Fabric samples were exposed to the harsh tropical environment of Panama.
Data accessibility	Raw data of shotgun metagenomics of microbial communities on plastic fabric materials have been deposited in MG-RAST (https://www.mg-rast.org/mgmain.html?mgpage=project&project=mgp85570) and can be retrieved using accession numbers: mgm4794685.3–mgm4794690.3.
Related research article	O. Radwan, J. S. Lee, R. Stote, K. Kuehn, O. N. Ruiz. Metagenomic Characterization of Microbial Communities on Plasticized Fabric Materials Exposed to Harsh Tropical Environments. International Biodeterioration & Biodegradation **154**, 2020, 105061.

## Value of the Data

• Raw metagenomic data of microbial communities could be an asset dataset to provide genomic information related to the structure and composition of microbial communities associated with biodegraded plastic fabric materials.
• Draft genomes identified from the dataset can be used to understand the underlying mechanisms by which microorganisms biodegrade plastics, and may help in development of biodegradation resistant materials and new plastic bioremediation approaches.
• These metagenomic data are valuable genomic sources for comparative metagenomics and can be exploited as a reference for other research teams interested in better understanding pathways and mechanisms involved in biodeterioration of plastic materials.
• Functional annotation of sequenced reads from the six different plastic fabric materials will help in elucidating the true composition and behavior of the complex microbiomes associated with environmentally exposed fabrics.

## 1 Data Description

**Fig. 1:**
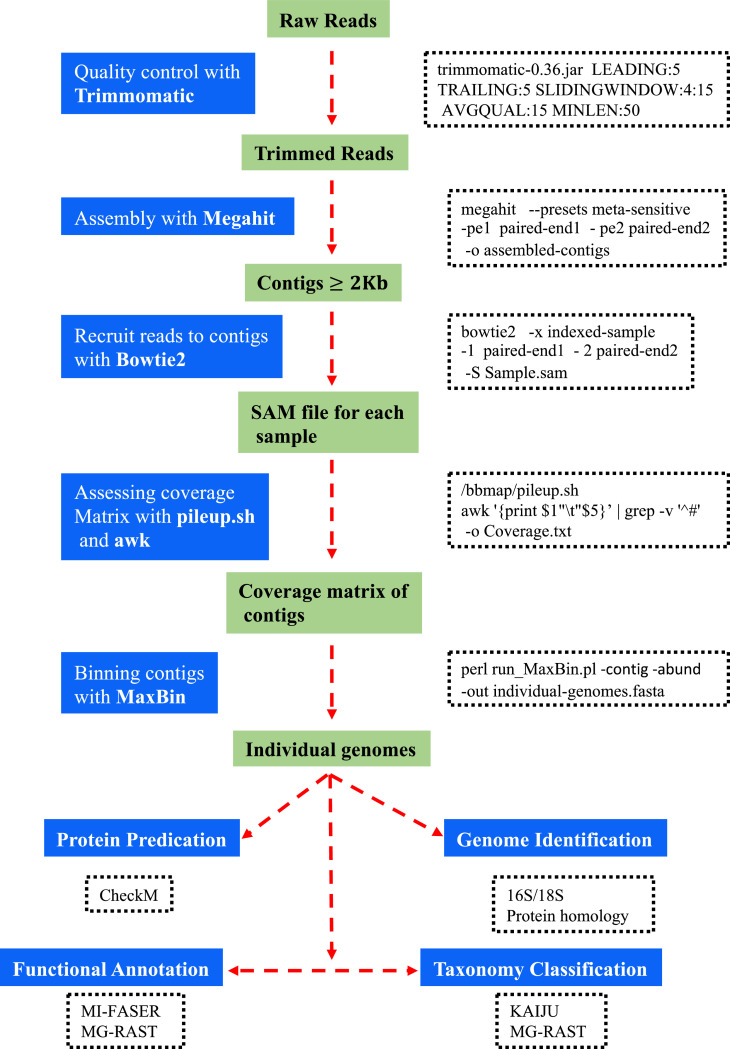
Schematic summary of the bioinformatics pipeline used to analyze shotgun metagenomic datasets.

**Fig. 2:**
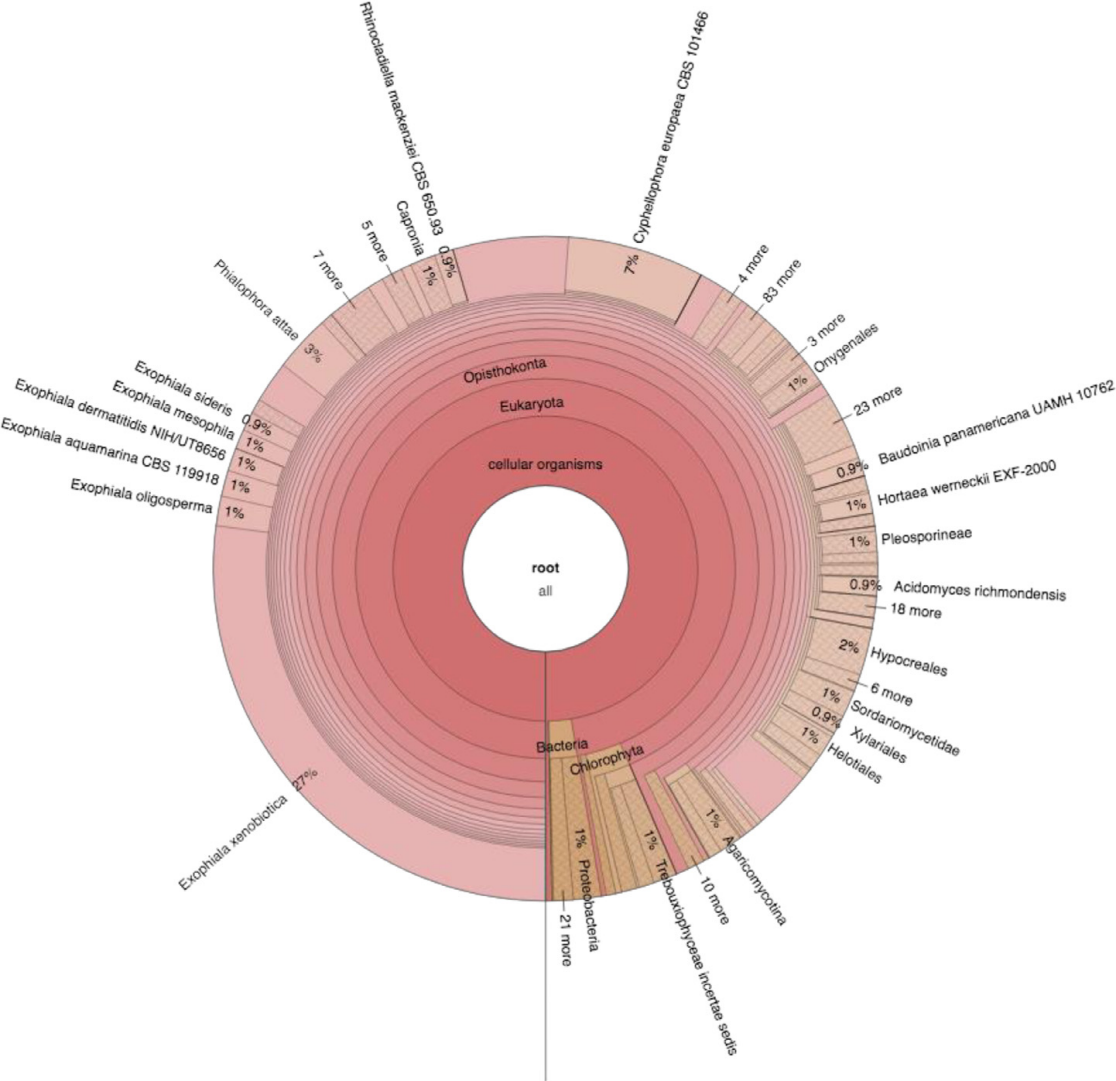
Diagram of the microbial composition and classification of the microbiome associated with fabric sample A developed by the KIAJU database.

**Fig. 3:**
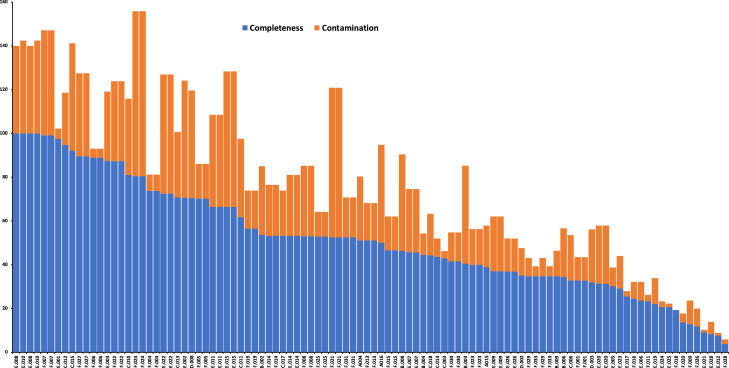
Percent of completeness and contamination of each microbial genome generated by CheckM bioinformatic program.

The datasets presented in this article are the raw sequences of pair-end reads with 100 bp length generated by Illumina HiSeq2000 platform. Shotgun metagenomics of six plastic fabric materials exposed to a harsh tropical environment produced 1.61 Gb of raw reads with a total of 161 Gb of 100 bp sequences [1]. The data files in FASTQ format were deposited in MG-RAST (https://www.mg-rast.org/) and can be retrieved using accession numbers: mgm4794685.3–mgm4794690.3. In this article, Fig. 1 provides a summary of the in-house pipeline that was established for bioinformatics analysis of metagenomic data. Table 1, contains a summary of raw reads, trimmed reads and total sequences (bp) from each sample. Table 1 also presents the number of sequences after trimming and the percent of surviving reads compare with the raw reads. Surviving reads from paired-end are reads after applying the trimming procedure. Table 2 summarizes the results of genomic assembled contigs generated by the MEGAHIT assembler program using trimmed sequences from the six fabrics. The sum (Mb), number of contigs > 500 bp, L50, N50 and the longest contig from each sample are presented in Table 2. N50 is the number of contigs whose length when summed up covers 50% or more of the genome assembly while L50 is the length of the smallest contig in the N50 set. Fig. 2 shows an overall summary of microbial distribution and taxon paths in one of the six plastic fabric materials. The data shown in Fig. 2 have been generated by KAIJU [2], a bioinformatic pipeline that is rapid and sensitive for taxonomic classification of short predicted proteins from metagenomic reads. Tables 3–8 summarize the results from MaxBin analysis that provided the different microbial genomes in each of the six plastic fabrics that were exposed to a harsh tropical environment for 14 months. Those microbial genomes are initially classified to different species of algae, black yeast, fungi and bacteria using KAIJU [2]. Tables 3–8 also show the genome size (Mb), GC content, classification and genome identification for each identified microbial genome. Fig. 3 shows the percent of completeness and contamination of each microbial genome presented in Tables 3–8 calculated using CheckM bioinformatic program [3]. A functional annotation summary of proteins predicted with MG-RAST from the sequenced reads is presented in Table 9. Table 9 shows twenty-eight functional categories with Carbohydrates; Amino Acids and Derivatives; Protein Metabolism; Cofactors, Vitamins, Prosthetic Groups, Pigments; and Respiration being the foremost categories in the sequences from the six platic fabric samples.

**Table 1:** Summary of raw reads, trimmed reads, and total sequence reads (bp) from each sample. Also, the percentage of surviving pair end reads after applying the trimming procedure is provided.

	Raw reads	Sequence (bp)	Trimmed reads	% Surviving reads
**Sample A**	290,535,652	29,191,462,169	273,136,734	94.01
**Sample B**	242,732,424	24,394,813,486	228,956,282	94.32
**Sample C**	280,501,300	28,198,140,693	257,866,956	91.93
**Sample D**	283,123,548	28,452,082,418	261,796,312	92.47
**Sample E**	245,775,804	24,706,114,436	223,055,718	90.76
**Sample F**	264,529,024	26,594,662,007	247,595,802	93.60
**Total**	1,607,197,752	161,537,275,209	1,492,407,804

**Table 2.** Summary of sequence assembly from the different samples using Megahit assembly program.

Sample	Sum (Mb)	# Contigs > 500 bp	L50	N50	Max (bp)*
Sample F⁎⁎	1128	787,368	106,600	1902	184,053
Sample D	1120	611,503	57,474	3290	584,409
Sample A	1052	589,392	59,676	3150	977,595
Sample C	1006	718,706	105,794	1774	126,842
Sample B	925.5	492,053	52,173	3251	1,435,925
Sample E	802	615,666	11,915	1544	740,880
Total	6033.5	3,814,688			

N50 is the number of contigs whose length when summed up covers 50% or more of the genome assembly.

L50 is the length of the smallest contig in the N50 set.

⁎ The longest contig (bp) in each sample.

⁎⁎ Samples are ordered descending based on to their sum (Mb).

**Table 3.** Summary of MaxBin results showing the different genomes belonging to algae, black yeast, fungi, and bacteria from fabric sample A.

Genome code	Genome size (Mb)	GC content	Classification	Genome identification
B05	4.865	35.7	Algae	Watanabea sp.
B09	3.511	51.2	Algae	Coccomyxa sp.
B01	0.63	29	Black yeast	Exophiala sp.
B02	2.69	43.9	Black yeast	Rhinocladiella sp.
B03	3.212	49.7	Black yeast	Baudoinia sp.
B04	2.212	54.4	Black yeast	Cyphellophora sp.
B06	2.83	45.8	Black yeast	Exophiala sp.
B07	1.89	57.4	Black yeast	Cyphellophora sp.
B08	4.20	53.8	Black yeast	Phialophora sp.

**Table 4.** Summary of MaxBin results showing the different genomes belonging to algae, black yeast, fungi, and bacteria from fabric sample B.

Genome code	Genome size (Mb)	GC content	Classification	Genome identification
A18	29.31	51.1	Algae	Coccomyxa sp.
A20	17.33	50.4	Algae	Coccomyxa sp.
A14	66.73	46.5	Algae	Coccomyxa sp.
A15	28.91	55.1	Algae	Coccomyxa sp.
A16	26.21	56.1	Algae	Coccomyxa sp.
A05	0.13	41.1	Algae	Trebouxia sp.
A02	0.17	38.4	Algae	Watanabea sp.
A06	0.16	33.7	Algae	Watanabea sp.
A07	0.16	40.4	Algae	Watanabea sp.
A11	19.52	49.9	Black yeast	Baudoinia sp.
A19	13.29	46.2	Black yeast	Cladophialophora sp.
A09	10.95	48.1	Black yeast	Cyphellophora sp.
A10	10.03	54	Black yeast	Cyphellophora sp.
A08	0.16	42.4	Black yeast	Exophiala sp.
A03	0.24	43.1	Black yeast	Exophiala sp.
A01	0.13	32.5	Black yeast	Exophiala sp.
A04	33.34	51.6	Black yeast	Exophiala sp.
A17	23.88	52.6	Black yeast	Hortaea sp.
A12	17.27	55.1	Black yeast	Phialophora sp.
A13	17.95	65.1	Fungi	Melampsora sp.

**Table 5.** Summary of MaxBin results showing the different genomes belonging to algae, black yeast, fungi, and bacteria from fabric sample C.

Genome code	Genome size (Mb)	GC content	Classification	Genome identification
C03	2583.65	65.8	Acidobacteria	Granulicella sp.
C04	2445.965	60.9	Acidobacteria	Bryocella sp.
C13	4205.182	64.9	Acidobacteria	Terriglobus sp.
C14	2733.709	60.7	Acidobacteria	Terriglobus sp.
C18	3185.587	64.8	Acidobacteria	Terriglobus sp.
C12	4845.024	71.5	Actinobacteria	Jatrophihabitans sp.
C16	11235.87	46.8	Algae	Coccomyxa sp.
C07	5678.483	38.3	Algae	Watanabea sp.
C01	3120.5	28.9	Black yeast	Zasmidium sp.
C02	29277.454	56.9	Black yeast	Cyphellophora sp.
C05	386.73	52.2	Black yeast	Verruconis sp.
C06	14141.935	49.7	Black yeast	Cyphellophora sp.
C08	3408.081	60.2	Black yeast	Verruconis sp.
C09	9494.505	45.7	Black yeast	Neonectria sp.
C10	5638.762	72	Proteobacteria	Gluconacetobacter sp.
C11	4430.702	68.5	Proteobacteria	Methylobacterium sp.
C15	4963.268	72.6	Proteobacteria	Caulobacteraceae sp.
C17	3677.465	68.7	Proteobacteria	Methyloferula sp.
C19	1212.841	65.6	Proteobacteria	Methylobacterium sp.
C20	934.412	64.4	Proteobacteria	Methyloferula sp.

**Table 6.** Summary of MaxBin results showing the different genomes belonging to algae, black yeast, fungi, and bacteria from fabric sample D.

Genome code	Genome size (Mb)	GC content	Classification	Genome identification
D02	5.96	61.3	Acidobacteria	Terriglobus sp.
D10	1.43	65	Acidobacteria	Terriglobus sp.
D06	0.69	68.9	Actinobacteria	Jatrophihabitans sp.
D03	31.56	46.5	Black yeast	Cyphellophora sp.
D04	5.73	56.1	Black yeast	Verruconis sp.
D05	7.14	56.4	Black yeast	Cyphellophora sp.
D01	2.56	60.7	Fungi	Melampsora sp.
D08	19.60	40.1	Fungi	Ceraceosorus sp.
D07	0.75	68.7	Proteobacteria	Methylobacterium sp.
D09	0.97	67.5	Proteobacteria	Methylobacterium sp.

**Table 7.** Summary of MaxBin results showing the different genomes belonging to algae, black yeast, fungi, and bacteria from fabric sample E.

Genome code	Genome size (Mb)	GC content	Classification	Genome identification
E02	6.31	71	Actinobacteria	Williamsia sp.
E03	5.69	74.7	Actinobacteria	Jatrophihabitans sp.
E04	1.36	74.6	Actinobacteria	Actinomycetospora sp.
E05	1.40	74.8	Actinobacteria	Actinomycetospora sp.
E06	4.39	77.6	Actinobacteria	Actinomycetospora sp.
E07	7.24	71.9	Actinobacteria	Actinomycetospora sp.
E13	12.37	74.2	Actinobacteria	Geodermatophilus sp.
E14	5.27	71.6	Actinobacteria	Jatrophihabitans sp.
E15	5.96	71.9	Actinobacteria	Actinomycetospora sp.
E19	3.62	67.3	Actinobacteria	Jatrophihabitans sp.
E20	2.72	69.8	Actinobacteria	Actinomycetospora sp.
E21	2.67	69	Actinobacteria	Geodermatophilus sp.
E17	8.85	65.3	Alphaproteobacteria	Methylobacterium sp.
E01	7.99	48.9	Bacteroidetes	Spirosoma sp.
E11	11.70	69.1	Bacteroidetes	Parafilimonas sp.
E12	2.50	29.1	Black yeast	Zasmidium sp.
E16	0.95	44.9	Black yeast	Pyrenochaeta sp.
E18	1.53	47.4	Black yeast	Cyphellophora sp.
E23	8.95	57.2	Black yeast	Hortaea sp.
E26	12.28	44.8	Black yeast	Exophiala sp.
E27	1.67	52.8	Black yeast	Baudoinia sp.
E28	1.34	51.9	Black yeast	Dothistroma sp.
E08	4.95	65.7	Proteobacteria	Methylobacterium sp.
E09	4.84	73.8	Proteobacteria	Methylobacterium sp.
E10	8.26	42.3	Proteobacteria	Methylobacterium sp.
E22	5.79	68.8	Proteobacteria	Aureimonas sp.
E24	9.86	60.5	Proteobacteria	Methylobacterium sp.
E25	1.08	44.9	Acidobacteria	Acidobacterium sp.

**Table 8.** Summary of MaxBin results showing the different genomes belonging to algae, black yeast, fungi, and bacteria from fabric sample F.

Genome code	Genome size (Mb)	GC content	Classification	Genome identification
F04	6.98	66.9	Actinobacteria	Gordonia sp.
F05	4.59	71.3	Actinobacteria	Williamsia sp.
F08	3.44	74.1	Actinobacteria	Actinomycetospora sp.
F15	4.86	74.9	Actinobacteria	Actinomycetospora sp.
F18	2.31	71.1	Actinobacteria	Actinomycetospora sp.
F23	4.65	69.5	Actinobacteria	Nakamurella sp.
F25	2.81	71.1	Actinobacteria	Micrococcales sp.
F31	1.91	70.6	Actinobacteria	Jatrophihabitans sp.
F01	39.98	51.3	Alage	Coccomyxa sp.
F02	5.33	37.1	Alage	Watanabea sp.
F06	3.89	35.6	Bacteroidetes/	Parafilimonas sp.
F07	6.04	51.5	Bacteroidetes	Mucilaginibacter sp.
F27	6.53	44.7	Bacteroidetes	Parafilimonas sp.
F03	2.20	27.3	Black yeast	Cladophialophora sp.
F10	1.07	33	Black yeast	Zasmidium sp.
F11	7.73	42.6	Black yeast	Cyphellophora sp.
F17	0.31	39	Black yeast	Coniochaeta sp.
F21	26.81	57.8	Black yeast	Hortaea sp.
F22	22.34	55.3	Black yeast	Cyphellophora sp.
F26	5.31	52.3	Black yeast	Hortaea sp.
F32	0.65	44.9	Black yeast	Hortaea sp.
F09	0.74	30.5	Chlorophyta	Cephaleuros sp.
F28	0.58	26.6	Chlorophyta	Cephaleuros sp.
F12	16.85	72.9	Proteobacteria	Methylobacterium sp.
F13	4.23	69.7	Proteobacteria	Methylobacterium sp.
F14	3.23	70.7	Proteobacteria	Methylobacterium sp.
F16	1.21	66.5	Proteobacteria	Methylobacterium sp.
F19	2.91	69.2	Proteobacteria	Sphingomonas sp.
F20	0.81	67.1	Proteobacteria	Methylobacterium sp.
F24	10.18	66.7	Proteobacteria	Xylophilus sp.
F29	1.73	65	Proteobacteria	Sphingomonas sp.
F30	5.67	62	Proteobacteria	Methylobacterium sp.
F33	4.16	61.4	Proteobacteria	Rhodospirillales sp.

**Table 9.** Functional annotation showing the percentage of sequence reads containing predicated proteins of known functions.

Functional categories	A	B	C	D	E	F	Average
Carbohydrates	15.18	16.22	15.87	16.03	15.55	15.78	15.77
Amino acids and derivatives	12.92	14.33	11.48	12.61	11.24	11.28	12.31
Protein metabolism	10.32	9.20	8.02	8.12	6.44	6.72	8.14
Cofactors, vitamins, prosthetic groups, pigments	7.52	7.29	6.78	6.88	6.83	6.72	7.00
Respiration	8.11	9.02	4.64	5.95	3.23	3.45	5.73
Fatty acids, lipids, and isoprenoids	4.40	4.80	4.16	4.56	4.83	4.56	4.55
RNA metabolism	4.98	4.59	4.74	4.65	3.90	4.17	4.50
Nucleosides and nucleotides	3.21	3.37	2.78	3.12	2.45	2.52	2.91
Stress response	2.85	2.99	2.85	2.85	2.69	2.61	2.81
Metabolism of aromatic compounds	2.76	3.29	1.98	2.29	2.20	2.14	2.44
Cell wall and capsule	1.37	1.12	2.82	2.31	3.11	3.25	2.33
DNA metabolism	1.06	0.75	2.69	2.13	3.35	3.28	2.21
Virulence, disease and defense	0.96	0.75	2.14	1.75	2.49	2.39	1.75
Membrane transport	0.71	0.42	1.61	1.27	2.13	2.17	1.39
Sulfur metabolism	1.14	1.10	1.09	1.08	1.22	1.17	1.13
Regulation and cell signaling	0.69	0.58	1.03	0.93	1.15	1.14	0.92
Cell division and cell cycle	0.74	0.65	0.91	0.84	0.97	0.93	0.84
Phosphorus metabolism	0.39	0.35	0.81	0.68	1.03	0.95	0.70
Nitrogen metabolism	0.58	0.56	0.65	0.65	0.91	0.77	0.69
Photosynthesis	1.87	1.10	0.46	0.15	0.17	0.24	0.67
Phages, prophages, transposable elements, plasmids	0.33	0.18	0.79	0.63	1.10	0.95	0.66
Secondary metabolism	0.81	1.08	0.51	0.68	0.37	0.37	0.64
Motility and chemotaxis	0.15	0.03	0.80	0.60	0.91	0.86	0.56
Iron acquisition and metabolism	0.16	0.14	0.42	0.38	0.73	0.72	0.43
Potassium metabolism	0.12	0.09	0.30	0.24	0.37	0.38	0.25
Dormancy and sporulation	0.02	0.01	0.10	0.07	0.13	0.13	0.08
Clustering-based subsystems	9.39	8.65	12.08	11.12	13.40	13.13	11.30
Miscellaneous	7.26	7.34	7.48	7.41	7.08	7.22	7.30

## 2 Experimental design, materials and methods

### 2.1 Samples and exposure environments

The U.S. Army Research, Development and Engineering Center (Natick, MA) provided six plastic fabric materials after 14 months of exposure to harsh tropical environment in the Republic of Panama [1]. The plastic fabric samples were used for DNA extraction, library preparation of genomic DNA, high-throughput sequencing, and bioinformatic analysis.

### 2.2 Library preparation and DNA sequencing for metagenomic study

DNA from the six plastic fabric materials was extracted with the Qiagen DNeasy UltraClean Microbial extraction kit (Cat# 12224-250), and then used for library preparation and DNA sequencing. A 300 ng of DNA from each fabric sample was used for the preparation of the genomic library using the PrepX DNA Library kit and Apollo 324 NGS automatic library prep system (WaferGen, Fremont, CA). A high-throughput sequencing of TruSeq paired-end libraries was conducted using a whole-genome shotgun (WGS) approach on an Illumina HiSeq2000 platform generating 100 bp reads. A TruSeq SBS kit v3 for 2 × 101 cycles of Incorporation Reagent (ICR) was used for read sequencing (Illumina, Inc. San Diego, CA).

### 2.3 Bioinformatics analysis for metagenomics study

An in-house multifaceted bioinformatics pipeline (Fig. 1) was established for the stepwise processing of sequence data required for completion of the metagenomic study. Quality control of raw reads was performed by Trimmomatic version 0.36 [4], which allowed trimming low quality reads and short reads from raw reads. Trimmed reads were sorted by BBtools (https://jgi.doe.gov/data-and-tools/bbtools/) “bbnorm.sh” to ensure the compatibility and normalization of paired-end before mapping to different contigs using MEGAHIT assembler program [5]. Bowtie2 [6] was employed for mapping raw reads to contigs produced by MEGAHIT, and the BAM file from each fabric sample was used for generating the coverage matrix and abundance files. Binning of individual genomes in each fabric sample was performed by MaxBin bioinformatic program [7] using the abundance file and fasta contigs generated by MEGAHIT.

### 2.4 Functional annotation of metagenomic reads and genome identification

The functional annotation of metagenomic reads of each fabric sample exposed to the tropical environment was extracted from the MG-RAST analysis (https://www.mgrast.org/mgmain.html?mgpage=project&project=mgp85570). Both RNAmmer [8] and CheckM [3] programs were used for ribosomal RNA identification of each binned genome generated by the MaxBin bioinformatic program. KAIJU [2], a fast and sensitive bioinformatic pipeline, was used for taxonomic classification of predicted proteins from metagenomic reads. Additionally, CheckM was used for assessing the completeness and presence contamination of microbial genomes generated by MaxBin.

## Declaration of Competing Interest

The authors declare that they have no known competing financial interests or personal relationships that could have appeared to influence the work reported in this paper.
